# Going Solo: Discovery of the First Parthenogenetic Gordiid (Nematomorpha: Gordiida)

**DOI:** 10.1371/journal.pone.0034472

**Published:** 2012-04-18

**Authors:** Ben Hanelt, Matthew G. Bolek, Andreas Schmidt-Rhaesa

**Affiliations:** 1 Center for Evolutionary and Theoretical Immunology, Department of Biology, University of New Mexico, Albuquerque, New Mexico, United States of America; 2 Department of Zoology, Oklahoma State University, Stillwater, Oklahoma, United States of America; 3 Zoological Museum and Institute, Biocenter Grindel, University of Hamburg, Hamburg, Germany; Institut national de la santé et de la recherche médicale - Institut Cochin, France

## Abstract

Despite the severe fitness costs associated with sexual reproduction, its persistence and pervasiveness among multicellular organisms testifies to its intrinsic, short-term advantages. However, the reproductive assurance hypothesis predicts selection favoring asexual reproduction in sparse populations and when mate finding is difficult. Difficulties in finding mates is especially common in parasites, whose life cycles involve multiple hosts, or being released from the host into the external environment where the parasite can find itself trapped without a sexual partner. To solve this problem and guarantee reproduction, parasites in numerous phyla have evolved reproductive strategies, as predicted by the reproductive assurance hypothesis, such as hermaphroditism or parthenogenesis. However, this type of strategy has not been reported from species in the phylum Nematomorpha, whose populations have often been described as sparse. A new Nematomorpha species, *Paragordius obamai* n. sp., was discovered from Kenya, Africa, and appears to have solved the problem of being trapped without a mate by eliminating the need for males. *Paragordius obamai* n. sp. represents the first and only known species within this phylum to reproduce asexually. To determine the mechanism of this mating strategy, we ruled out the involvement of reproduction manipulating endosymbionts by use of next generation sequencing data, thus suggesting that parthenogenesis is determined genetically and may have evolved as a means to assure reproduction. Since this new parthenogenetic species and a closely related gonochoristic North American congener, *P. varius*, are easy to propagate in the laboratory, these gordiids can be used as model systems to test hypotheses on the genetic advantages and disadvantages of asexual reproduction and the genetic determinants of reproductive strategies in parasites.

## Introduction

Explaining the widespread and almost ubiquitous occurrence of sexual reproduction [1], throughout the plant and animal kingdoms, remains one of the greatest challenges for biologists, and has been called the “queen of problems in evolutionary biology” [2]. The costs associated with sexual reproduction can be severe and include the break-up of successful genetic combinations, the dilution of female's genetic contribution (two fold cost of sex [3], [4]), and the energetic cost and danger associated with mate finding and copulation. However, the pervasiveness of sexual reproduction suggests that these costs are outweighed by powerful factors having intrinsic, short-term advantages. These advantages include the purging of deleterious mutations [5], accelerated adaptation to changing environments and/or parasites (Red Queen Hypothesis [2]), and a reduction in selective interference among deleterious mutations in finite populations [6].

Despite the advantages of sexual reproduction, the reproductive assurance hypothesis predicts that parthenogenesis is favored in sparse populations where mates are difficult to find [7], [8]. Mate finding difficulty is especially exemplified by many parasite species, whose life cycles require movement of individuals between hosts or between hosts and the external environment. Although parasites have evolved numerous strategies to ensure successful movement between life cycle stages, completion of the life cycle is not sufficient if not accompanied in parallel by a member of the opposite sex; otherwise the parasite finds itself trapped in an environment without a sexual partner. To solve this problem and guarantee reproduction, parasites in numerous phyla have evolved reproductive strategies as predicted by the reproductive assurance hypothesis, such as hermaphroditism or parthenogenesis, allowing trapped individuals to reproduce [9]. However, up to now, parasites within the phylum Nematomorpha did not appear to have evolved a mechanism to assure reproduction.

**Figure 1:**
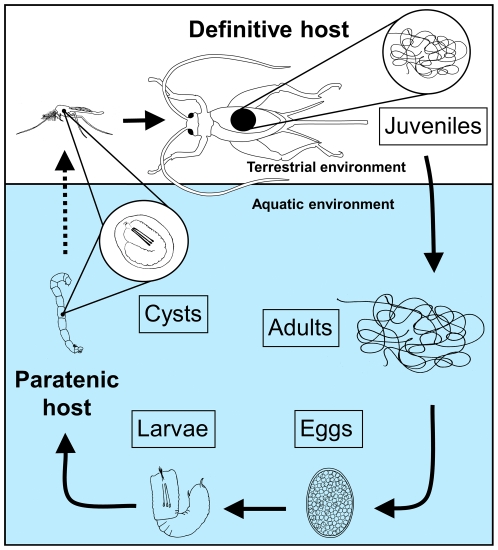
Typical gordiid life cycle. Gordiid adults normally pair and mate in aquatic environments such as rivers, streams, lakes, and ponds. Ovipositioning begins within hours to days and can last as long as 2–3 weeks and produces several million eggs. Larvae hatch within 10–14 days, are non-buoyant, and do not swim. Upon consumption, using their proboscis, larvae penetrate and encyst within a wide variety of aquatic animals, including fish, snails, crustaceans, and aquatic insect larvae. Of these hosts, only aquatic insect larvae transport the cysts to the terrestrial environment after metamorphosis (dashed line), where the hosts and the cysts are consumed by the insect definitive host. Many hosts containing cysts, such as snails, which are not normally consumed by the definitive host, are considered dead-end hosts. Within the definitive host, cysts excyst and the larvae penetrate and develop within the hemocoel. Upon maturation, worms manipulate host behavior to jump into water, enabling the worms to make a successful transition from the terrestrial to the aquatic environment.

The Nematomorpha is the sister phylum to the Nematoda [10]. Although these phyla share many features such as their general body shape, color, cuticle, and body organization, several key differences delineate these phyla. First, all members of the Nematomorpha are parasites as juveniles but free-living as adults. Second, due to their parasitic lifestyle, the nematomorph gut is largely non-functional, and juveniles feed by absorbing materials directly through their cuticle [11], [12], [13]. Freshwater nematomorphs (Nematomorpha: Gordiida), or gordiids, are a unusual group of parasites that mate and oviposit outside of their hosts [14]. Members of this phylum are strongly sexually dimorphic and until now have been described as dioecious. The unique gordiid life cycle (Fig. 1) involves transition of parasites within terrestrial arthropod hosts to free–living aquatic adults, which is partially achieved by parasite manipulation of hosts to commit ‘suicide’ by jumping into water [15], [16] triggering worms to escape. Freshwater gordiids use various definitive hosts including hemimetabolous insects such as orthopterans (crickets, grasshoppers, and locusts), cockroaches, and mantids, and holometabolous insects such as beetles [17].

Once released from their hosts, male and female gordiids pair and mate, often forming large Gordian knots [18] especially in isolated waters such as vernal pools, desert springs, and small, low-flowing streams, which act naturally to concentrate individuals in these aquatic microhabitats. However, since gordiids rely on large and long river systems and areas with vast aquatic habitats, they must overcome the problem of release by the host distantly in space and time, which can result in worms finding themselves trapped in aquatic systems without mates.

Here we report on the discovery of a new *Paragordius* species, which has solved the problem of mate-finding by the elimination of males. *Paragordius obamai* n. sp. represents the first and only known species within this phylum to reproduce asexually. To gain a better understanding of its reproductive strategy, we tested whether parthenogenesis in *P. obamai* n. sp. is driven by the presence of reproduction manipulation endosymbionts. In addition we discuss the possibility that parthenogenesis could be driven by reproductive assurance and could be much more common in gordiids than previously thought.

## Results

### Taxonomy

Genus: *Paragordius* Camerano, 1897

***Paragordius obamai*** n. sp.

urn:lsid:zoobank.org:act:65BDF132-3996-4F4C-80A3-573C074406E7

**Etymology.** This species is named in honor of the 44^th^ president of the United States of America, Barack H. Obama, whose father was raised and paternal step-grandmother resides in Nyang'oma Kogelo (Kogelo), Kenya, 19 km northwest of the type locality.

**Holotype.** Female in ZMH (Zoological Museum, Hamburg, Germany), accession number ZMH V13265 reared from a cyst from a *Biomphalaria pfeifferi* snail collected at Kasabong stream, a small stream in Nyanza province, Kenya (−0.1519°, 34.3355°, approx. 1170 m altitude), which flows into Winam Bay of Lake Victoria, 2 km to the south.

**Paratypes.** Two females in ZMH, accession number ZMH V13266–V13267, and one female accession number MSBPARA940 in the Museum of Southwestern Biology Parasitology Division, University of New Mexico (USA); all worms were reared from cysts from *B. pfeifferi* snails collected at Kasabong stream.

**Distribution.** Lake Victoria Basin, western Kenya, Africa. In addition to the type locality, cysts have also been found in Asao stream, Nyanza province, Kenya (−0.3176°, 35.0065°, approx. 1250 m altitude), which flows into Winam Bay of Lake Victoria, 17 km to the west, and is located 65 km southeast of the type locality.

**Hosts.** Paratenic host was *B. pfeifferi*, but likely includes numerous other stream vertebrates and invertebrates [19]. Natural definitive insect host is unknown; in the laboratory, *Acheta domesticus*, *Gryllus texensis*, and *Gryllus firmus* crickets served as definitive hosts.

**Figure 2:**
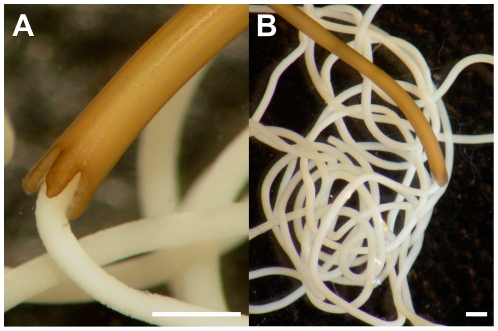
*Paragordius obamai* n. sp. female laying eggs. Female worm (brown) in the process of laying egg strings (white) containing eggs: (A) showing trilobed posterior end; (B) a worm and about 15% of total lifetime egg production. Scale bars = 2 mm.

**Figure 3:**
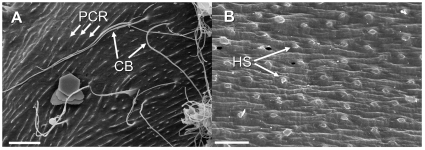
*Paragordius obamai* n. sp. cuticle. (A) longitudinal ridges, from which long cuticular bristles emerge on the inside of the tail lobes, and (B) hemispherical structures on the mid-body surface. Abbreviations: PCR, parallel cuticular ridges; CB, cuticular bristles; HS, hemispherical structures. Scale bars = 10 µm.

**Description.** *Adults:* light brown to yellow in color, 15.0 (10.9–23.2) cm long by 1.4 (0.9–1.8) mm wide. The posterior end is trilobed (Fig. 2A), with the dorsal lobe being longer (400 µm) and broader (275 µm) than the lateroventral lobes (380×220 µm). The cloacal opening is is positioned centrally between the three lobes. Inner surfaces of lobes contain longitudinal, parallel cuticular ridges (Fig. 3A) unique to this species, which likely aid in directing the movement of egg string during ovipositing (Figs. 2A, B). Long cuticular bristles emerge from these ridges. Cuticle contains small hemispherical structures (Fig. 3B), varying slightly from other *Paragordius* species.

*Eggs and Egg strings:* Egg strings, 276 (204–391) µm wide, were deposited in long uninterrupted strings (Fig. 2B). Females did not attempt to attach egg strings to aerators, air hoses, or 1 liter bottles but instead deposited egg strings free in the water column, as previously described for *P. varius* [20]. Undeveloped eggs were 41.6 (36.5–48.7) µm by 26.9 (23.4.0–30.2) µm. Larvae developed within 3–4 weeks.

**Figure 4:**
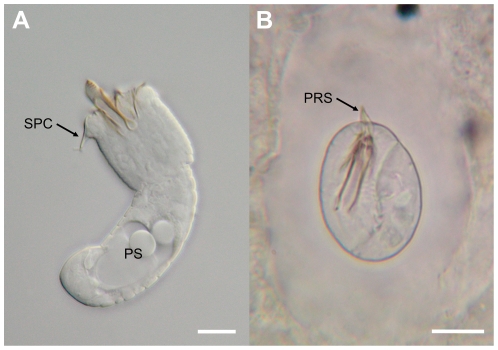
*Paragordius obamai* n. sp. (A) free living larva and (B) cyst within *Biomphalaria pfeifferi* snail. Abbreviations: PS, pseudointestine; SPC, spine in crown; PRS, protruding spines. Scale bars = 10 µm.

*Larvae:* Cylindrical body divided by a septum into two regions, the preseptum and postseptum (Fig. 4A). The preseptum measures 29.1 (24.5–31.9) µm by 12.7 (12.0–14.8) µm and contains an evertable proboscis 12.2 (9.9–14.1) µm by 3.3 (2.8–4.2) µm containing three internal stylets. The postseptum measures 36.1 (33.2–38.7) µm by 10.4 (8.4–11.7) µm and contains the pseudointestine 13.0 (8.4–15.9) µm by 7.0 (5.0–9.3) µm which is composed of two anterior granules and a large posterior mass. Externally, larvae are annulated and two pairs of spines are located on the posterior ventral portion of the postseptum.

*Cysts:* Larvae are folded within cysts (Fig. 4B) and measured 28.0 (24.5–32.4) µm by 20.0 (17.4–23.7) µm. The cyst wall was clear and its thickness measured 12.5 (8.9–17.1) µm by 9.5 (6.1–13.7) µm. Larvae, within completely formed cysts, had distinct spines protruding from their preseptum (Fig. 4B).

**Diagnosis.** Seventeen species of *Paragordius* are known: 9 from Africa, 5 from South America, 1 from Oceania, 1 from North America, Central America, and South America, and 2 from Europe (Table 1). All females show three tail lobes, characteristic for the genus, and all males show two tail lobes which are longer than twice their width. *Paragordius obamai* n. sp. is distinguished from other African species by the presence of hemispherical cuticular substructures of varying size (Fig. 3B) that differ from the polygonal structures called areoles present in other *Paragordius* species with the exceptions of *P. cinctus* and *P. somaliensis*. No cuticular structures have been described for *P. cinctus* [21]; whereas small substructures similar to those in *P. obamai* n. sp. have been described in *P. somaliensis* [22]. However, the dorsal tail lobe is distinctly longer in *P. obamai* n. sp. than in *P. somaliensis*.

**Table 1:** *Paragordius* spp. distribution and the status of male descriptions for each species.

Geographic region	Species	Describer	Males
Africa	*P. areolatus*	Linstow, 1906	No
	*P. cinctus*	Linstow, 1906	Yes
	*P. dartevellei*	Sciacchitano, 1958	No
	*P. laurae*	Sciacchitano, 1958	No
	*P. marlieri*	Sciacchitano, 1958	No
	*P. obamai* **n. sp.**	This paper	No
	*P. somaliensis*	Sciacchitano, 1962	No
	*P. tanganikensis*	Sciacchitano, 1958	No
Central America	*P. diversolobatus*	Heinze, 1935	No
	*P. flavescens*	Linstow, 1906	No
Europe	*P. stylosus*	(Linstow, 1883) Camerano, 1897	Yes
	*P. tricuspidatus*	(Dufour, 1828) Camerano, 1897	Yes
North and South America	*P. varius*	Leidy, 1851	Yes
	*P. andreasii*	Zanca and de Villalobos, 2006	Yes
South America	*P. esavianus*	Carvalho, 1942	Yes
	*P. minusculus*	Carvalho, 1944	Yes
	*P. mulungensis*	Sciacchitano, 1958	No
Oceania	*P. emeryi*	(Camerano, 1895) Camerano, 1895	No

The cuticular pattern of *P. obamai* resembles that of *P. varius* from North America [23], [24], and *P. tricuspidatus* from Europe [25]. However, *P. obamai* n. sp. differs from these species in the structure of the inner surfaces of the tail lobes. In *P. obamai* n. sp., the inner surfaces of the tail lobes contain longitudinal ridges, from which long cuticular bristles emerge (Fig. 3A). Many such ridges run in parallel from the cloacal opening to the terminal end of each lobe. In contrast, the inner surfaces of the tail lobes of *P. varius* are more or less smooth [23]; whereas those of *P. tricuspidatus* carry roundish or slightly elongate structures, from which long bristles emerge (Schmidt-Rhaesa, unpublished observations). These are oriented in a similar pattern as in *P. obamai* n. sp., but never fuse to form distinct ridges. Therefore, the observed combination of adult characters observed in *P. obamai n. sp.* differentiates this species from all other known *Paragordius* species.

### Laboratory rearing and histology

*Paragordius obamai* n. sp. was successfully established in 3 species of crickets from two genera (Table 2). Only female worms were released from the 30 infected crickets. Dissection of hosts, during the period of worm maturation, did not reveal any male worms. Histological sections of female adult worms revealed normal female anatomy. The body cavity contained paired gonads, within which oocytes and nurse cells were clearly visible (data not shown). Male reproductive organs, including the paired testes tubes, and sperm were not observed in any sections, and all internal morphological features were similar to that of *Paragordius varius* females [26], [27].

**Table 2:** Gender of *P. obamai* n. sp. which emerged from three species of cricket host in the laboratory.

Host		Worm Sex	
Species	Number	♀	♂
*Acheta domesticus*			
	1	1	0
	2	2	0
	3	1	0
	4	3	0
	5	2	0
	6	1	0
	7	1	0
	8	4	0
	9	3	0
	10	2	0
	11	2	0
	12	2	0
	13	3	0
	14	2	0
	15	3	0
	16	2	0
	17	4	0
	18	3	0
	19	1	0
	20	1	0
	21	2	0
	22	2	0
	23	1	0
	24	1	0
*Gryllus texensis*			
	25	1	0
	26	1	0
	27	1	0
	28	2	0
*Gryllus firmus*			
	29	1	0
	30	1	0
TOTAL		56	0

### 4-5-4 Pyrosequencing

A total of 27,087 bacterial 16S sequences resulted from the pyrosequencing of the DNA derived from the 5 *P. obamai* n. sp. life cycle stages. Of these, 2,295 low quality and short sequence reads were removed from further analysis (Table S1). The remaining sequences were ∼300 bp (125 to 530 bp) in length. MegaBLAST analyses placed several sequences within bacterial endosymbiont groups known to cause reproductive manipulation, including the genera of *Rickettsia*, *Arsenophonus*, and *Flavobacterium* (Table S1). *Rickettsia*-like sequences were found only in the DNA sample from eggs (*n* = 2) and larvae (*n* = 9, representing 8 unique sequences). BLAST analyses revealed these *Rickettsia*-like sequences to be highly similar to those obtained from bacteria collected as part of environmental samples from the gut of marine fish, human skin, biofilm mats of montane wetlands, and uranium waste water, etc. (Table S2).

*Flavobacterium*–like sequences were found only in the 8 week post infection (wpi) juvenile DNA sample (*n* = 21, representing 16 unique sequences). Similarly, *Arsenophonus* -like sequences were found only in the 3 wpi juvenile DNA sample (*n* = 20, representing 12 unique sequences). Subsequent BLAST analyses revealed that the *Flavobacterium* sp.-like sequences were most similar to uncultured bacteria collected from seawater and from an Alaskan oil well. The *Arsenophonus* sp.-like sequences also have been recovered from uncultured environmental samples, for example from a fish and beetle gut (Table S2), but also show high similarity to endosymbionts reported from a wasp (*Polistes nimpha*) and a nematode (*Steinernema diaprepesi*). However, many of the *Arsenophonus* spp. listed in GenBank need additional characterization and have not been implicated to be involved with reproduction alteration.

Phylogenetic analyses supported the findings of the BLAST analyses for *Rickettsia*-like sequences (Fig. S1), *Arsenophonus*-like sequences (Fig. S2), and Flavobacteria (Fig. S3). Each analysis clearly shows a much closer association between newly recovered sequences and their corresponding BLAST results than to the known reproductive manipulators, supporting the notion that many of these sequences were derived from environmental contaminants, and do not represent endosymbionts.

## Discussion

Our laboratory infections clearly demonstrated that *P. obamai* n. sp. is capable of reproducing asexually. In addition, our histological work showed normal female anatomy in adult worms and the lack of sperm within females, suggesting that reproduction occurs by parthenogenesis. The lack of male worms also suggested that the reproductive strategy of *P. obamai* n. sp. is by thelytokous parthenogenesis, the production of diploid female-only offspring from unfertilized eggs.

In a comprehensive review on the occurrence of parthenogenesis, Bell (1982) noted that all species in the phylum Nematomorpha are gonochoricistic and asexual reproduction has never been reported in the phylum. Since this review, laboratory experiments, field observations, and field isolations of numerous gordiid species have not produced evidence of asexual reproduction. For example, the North American species *P. varius* has been in laboratory culture for over a decade [28] with the production of equal sexes [29]. Furthermore, experimental infections of North American and Old World gryllid species with *P. varius* cysts from several populations within North America indicated that all of these populations produced male and female worms (M. Bolek, personal observations). In addition, our observations revealed that unmated *P. varius* females as well as unmated females of *Chordodes morgani*, *Gordius robustus*, and *Gordius difficilis* never produced viable eggs or oviposited egg strings (M. Bolek and B. Hanelt, personal observations), clearly indicating that mating is essential in other gordiid species.

Reproductive manipulation by parasites is common in invertebrates and occurs by one of four mechanisms: feminization of genetic males, cytoplasmic incompatibility, male killing, and parthenogenesis induction [30]. Induction of parthenogenesis usually results in thelytokous parthenogenesis as noted in many insects and some nematodes by endosymbionts such as *Wolbachia* spp. and *Rickettsia* spp. [31], [32]. Due to their close phylogenetic relationship with nematodes and intimate and extended contact with their arthropod hosts, gordiids have been hypothesized to be good candidates for reproduction altering endosymbionts. However, three gordiid species *Paragordius tricuspidatus*, *Spinochordodes tellinii*, and *Gordius robustus* have been tested for and found to be free of *Wolbachia* symbionts [33], [34]. *Gordius robustus* was found to lack internal bacterial symbionts altogether, and it was suggested that the feeding behavior of gordiids, directly through the cuticle during the juvenile parasitic phase [11], [12], [13], is responsible for their depauperate internal flora/fauna [33].

In the current study, we found the presence of a few endosymbionts associated with *P. obamai* n. sp., although none of these were closely allied to any known reproductive manipulating endosymbiont (Table S1). Because eggs and larvae of *P. obamai* n. sp. were maintained in non-sterile artificial spring water, it is highly likely that these bacteria represent environmental contaminants. Finding *Flavobacterium*-like and *Arsenophonus*-like sequences only in the developing *P. obamai* n. sp. juveniles dissected from *A. domesticus* crickets suggests that these bacteria likely originated from the cricket host and represent contaminants.

Since all reproduction altering endosymbionts are transmitted vertically, from mother to offspring, they as well as their 16S sequences should be present in all life cycle stages of *P. obamai* n. sp. [30]. The lack of endosymbionts within all 5 *P. obamai* n. sp. life cycle stages, as well as data from our BLAST and phylogenetic analyses, strongly suggest that the putative endosymbionts found as part of this study are contaminants from the worm's host or from the environment.

Thus, our sequencing data firmly supports the conclusion that reproductive manipulating endosymbionts of the five major known bacterial groups are not found in *P. obamai* n. sp. worms. Obviously this data cannot rule out the presence of unique and undetermined bacteria or other symbionts leading to parthenogenesis. However, based on current knowledge, we must conclude that parthenogenesis in *P. obamai* n. sp. is due to genetic mechanisms. Evidence capable of identifying the cytological mechanism of thelytoky (automixis v. apomixis) in *P. obamai* n. sp. is currently lacking. In order to distinguish between mitotic and meiotic parthenogenesis, studies involving cytology and genetic population structure are needed. Since adult worms are extremely difficult to locate in natural habitats, population studies will need to focus on cysts or adults grown from cysts in the laboratory.

As mentioned above, asexual reproduction is rare and in those cases where it occurs, we are challenged to explain the factors leading to its evolution. The reproduction assurance hypothesis provides a possible explanation of why *P. obamai* n. sp. evolved parthenogenesis. Gordiids normally mate as free-living adults in aquatic habitats after escaping from their arthropod host, and reproduction occurs in aquatic habitats. Studies of gordiids from 3 continents indicate that multiple infections are relatively rare, occurring in 8–18% of hosts [16], [35], [36]. These studies thus indicate that gordiids, which naturally occur as single infections and are released seasonally over a 1–3 month period in complex aquatic habitats, can often lead to low worm densities and mate finding difficulties. The ability of *P. obamai* n. sp. to undergo parthenogenesis could be a solution to the problem of mate finding and provides a mechanism for this species to ensure reproduction.

Although we are unable to explain why this species appears to be the only gordiid to have undergone selective pressures to become parthenogenetic, evidence from the literature suggest that the genus *Paragordius* may contain other species with a similar reproductive strategy. In fact, of the 18 known *Paragordius* species, males have only been described from 39% of these species (Table 1). In addition, of the 9 *Paragordius* species reported from Africa, males have been documented for only one species (*P. cinctus*). This is in contrast to 2 other widely distributed gordiid genera. Of the 82 valid species in the genus *Gordius*, males have been documented in 88% of these species [37]; whereas males have been documented in 65% of the 55 valid species in the genus *Chordodes* [38], [39]. Our observations on *P. obamai* n. sp. and the lack of male specimens from most species in the genus *Paragordius* raises the intriguing possibility that parthenogenesis is much more common in this group than previously recognized, and must be confirmed with additional field sampling and establishment of additional life cycles in the laboratory.

Finally, as mentioned above, a close congener to this new species, *P. varius* has also been domesticated [28]. Comparison of *P. obamai* n. sp. and *P. varius* will thus allow us to test various hypotheses about the advantages and disadvantages of sex in parasitic species, and may even allow us to probe the genetic determinants of reproductive strategies of closely related parasites.

## Materials and Methods

### Sample collection and experimental infection

*Biomphalaria pfeifferi* snails were collected from small streams in the Lake Victoria Basin, Kenya. Snails were screened for gordiid cysts following a previously published protocol [40]. Briefly, individual snails were crushed between microscope slides and the soft tissue was separated. Small pieces of soft tissue were placed, with a drop of water, onto a slide and flattened with a coverslip. Tissue was examined for cysts at 200× and 400×. Streams positive for cysts were searched for gordiid adults by sight and the use of a dip-net.

Live snails containing cysts were returned to the laboratory in the United States and fed to *Acheta domesticus*, *Gryllus texensis*, and *Gryllus firmus* crickets [28]. Starting at 4 wpi, crickets were placed in water daily to allow matured worms to exit. Worms emerging from hosts were immediately separated (if needed) and maintained in individual 1000 ml water bottles. Fifteen crickets were dissected and examined, at 3 and 8 weeks post infection, and after female worms emerged from their cricket hosts, for the presence of male *P. obamai* n. sp. worms.

### Microscopy and histology

Eggs, larvae and cysts were examined at 400× and 1,000×. At least 30 measurements were taken of each character, following a previously described protocol [20]. Adults were examined by scanning electron microscopy (SEM) by removing a 5–10 mm section of the mid-body cuticle and the entire posterior end. The cuticle and posterior ends were critically point dried, mounted on aluminum stubs, coated with gold palladium, and observations were made using a LEO SEM 1524 (Carl Zeiss, Jena, Germany) at 10 kV.

To examine adult morphology, two worms were processed for histological sectioning. Fresh worms were fixed in Bouin's fluid, sectioned at 5 µm using typical histological techniques [41], stained with Semichon's acetocarmine, counterstained with fast green, and mounted in Canada balsam.

### 4-5-4 Pyrosequencing and analyses

Total genomic DNA was extracted from 5 life cycle stages: 1) eggs, 2) larvae, 3) 3 week old juveniles, 4) 8 week old juveniles, and 5) adults. Juveniles were dissected from the hemocoel of *A. domesticus* cricket hosts. For each sample approximately 0.5–2.0 g of tissue was isolated and placed in 100% EtOH, dried at room temperature for 1–3 hours, and used for DNA extraction using the E.Z.N.A.® Mollusc DNA Kit (Omega Bio-tek, Norcross, Georgia) or the DNeasy® Blood and Tissue Kit (Qiagen, Hilden, Germany) following the manufacturer's instructions. Extracted DNA was stored at −70C. Bacterial tag-encoded FLX amplicon pyrosequencing (bTEFAP) was performed by the Research and Testing Laboratory (Lubbock, TX) as described previously [42], [43]. Initial generation of the sequencing library was done using universal 16S rDNA sequencing primers [44], 530f: GTG CCA GCM GCC GCG G, and 1100r: GGG TTG CGC TCG TTG targeting a region of about 570 bp. However, the length of this region can vary between bacterial species.

Following sequencing, failed, low quality, and short sequence reads (<125 bp), tags, primers, and non-16S bacterial reads were removed along with chimeras using Black Box Chimera Check (B2C2) employing default settings [45]. The identity of remaining bacterial sequences was determined by denoising and assembling them into clusters then querying them using a distributed MegaBLAST. NET algorithm [46] against a database of high quality 16s bacterial sequences derived from NCBI. Based on the percent query length aligning to a database sequence, bacteria were taxonomically classified by their identity scores to genera (3–5% divergence). Sequences identified as falling within genera of known reproductive manipulators were contig aligned using Sequencer version 5.0.1 (Gene Codes, Ann Arbor, Michigan). DNA sequences were compared to other sequences in the GenBank database using BLAST [47]. The 2 GenBank entries returning the highest similarity score were documented (Table S2). Sequences classified as *Rickettsia*-like, *Arsenophonus*-like, and Flavobacteria were further analyzed by comparing them to their closest BLAST match and reference sequences retrieved from GenBank using molecular phylogenetic analyses. Maximum likelihood method based on the Kimura 2-parameter model was used to produce unrooted trees in MEGA5 [48].

### Nomenclatural Acts

The electronic version of this document does not represent a published work according to the International Code of Zoological Nomenclature (ICZN), and hence the nomenclatural acts contained in the electronic version are not available under that Code from the electronic edition. Therefore, a separate edition of this document was produced by a method that assures numerous identical and durable copies, and those copies were simultaneously obtainable (from the publication date noted on the first page of this article) for the purpose of providing a public and permanent scientific record, in accordance with Article 8.1 of the Code. The separate print-only edition is available on request from PLoS by sending a request to PLoS ONE, 1160 Battery Street Suite 100, San Francisco, CA 94111, USA along with a check for $10 (to cover printing and postage) payable to “Public Library of Science”.

In addition, this published work and the nomenclatural acts it contains have been registered in ZooBank, the proposed online registration system for the ICZN. The ZooBank LSIDs (Life Science Identifiers) can be resolved and the associated information viewed through any standard web browser by appending the LSID to the prefix “http://zoobank.org/”. The LSID for this publication is: urn:lsid:zoobank.org:pub: 703CE118-461C-46A0-993F-2AD849DFCECB.

## Supporting Information

Figure S1 **Molecular Phylogenetic analysis by Maximum Likelihood method of** ***Rickettsia*** **spp.** The tree with the highest log likelihood (−1457.1543) is shown, and is drawn to scale, with branch lengths measured in the number of substitutions per site. The analysis involved 20 nucleotide sequences: reference *Rickettsia* samples from GenBank (red), sequences recovered from different life cycle stages of *P. obamai* n. sp. from this study (blue), and the 2 closest sequences recovered from GenBank BLAST hits (orange). All positions containing gaps and missing data were eliminated. There were a total of 305 positions in the final dataset. Clearly, the *Rickettsia* species known to induce parthenogenesis groups away from the sequences recovered as part of the current study and their closest BLAST match. Also note that these *Rickettsia*-like sequences were only recovered from two of the 5 life cycle stages tested. For additional information on sequences recovered by BLAST and contigs, see Table S1.
(TIF)

Figure S2 **Molecular Phylogenetic analysis by Maximum Likelihood method of** ***Arsenophonus*** **spp.** The tree with the highest log likelihood (−988.1006) is shown and is drawn to scale, with branch lengths measured in the number of substitutions per site. The analysis included 21 nucleotide sequences: reference *Arsenophonus* samples from GenBank (red), sequences recovered from 3 week old juvenile *P. obamai* n. sp. (blue), and the 2 closest sequences recovered from GenBank BLAST hits (orange) for each of the sequences recovered. All positions containing gaps and missing data were eliminated. There were a total of 321 positions in the final dataset. Note that the new sequences cluster with a sequence previously identified as *Budvicia* sp., as well as with symbionts known from wasps, not known to manipulate reproduction. Also note that these *Arsenophonus*-like sequences were only recovered from one of five life cycle stages tested. For additional information on sequences recovered by BLAST and contigs, see Table S1.
(TIF)

Figure S3 **Molecular Phylogenetic analysis by Maximum Likelihood method of Flavobacteria.** The tree with the highest log likelihood (−802.3099) is shown and is drawn to scale, with branch lengths measured in the number of substitutions per site. The analysis included 20 nucleotide sequences: reference Flavobacteria samples from GenBank (red), sequences recovered from 8 week old *P. obamai* n. sp. larvae (blue), and the 2 closest sequences recovered from GenBank BLAST hit (orange) for each sequence recovered. All positions containing gaps and missing data were eliminated. There were a total of 341 positions in the final dataset. Note that all new sequences and their closest BLAST matches fall into a separate group from the known Flavobacteria known to cause male killing. Also note that these Flavobacteria-like sequences were recovered from only one of five life cycle stages tested. For additional information on sequences recovered by BLAST and contigs, see Table S1.
(TIF)

Table S1 (DOCX)

Table S2 (DOCX)
