# Supplementary figures and images for: Going Solo: Discovery of the First Parthenogenetic Gordiid (Nematomorpha: Gordiida)
Source: PLoS One. 2012 Apr 18;7(4):e34472. doi: 10.1371/journal.pone.0034472 (PMC3329510; doi:10.1371/journal.pone.0034472)

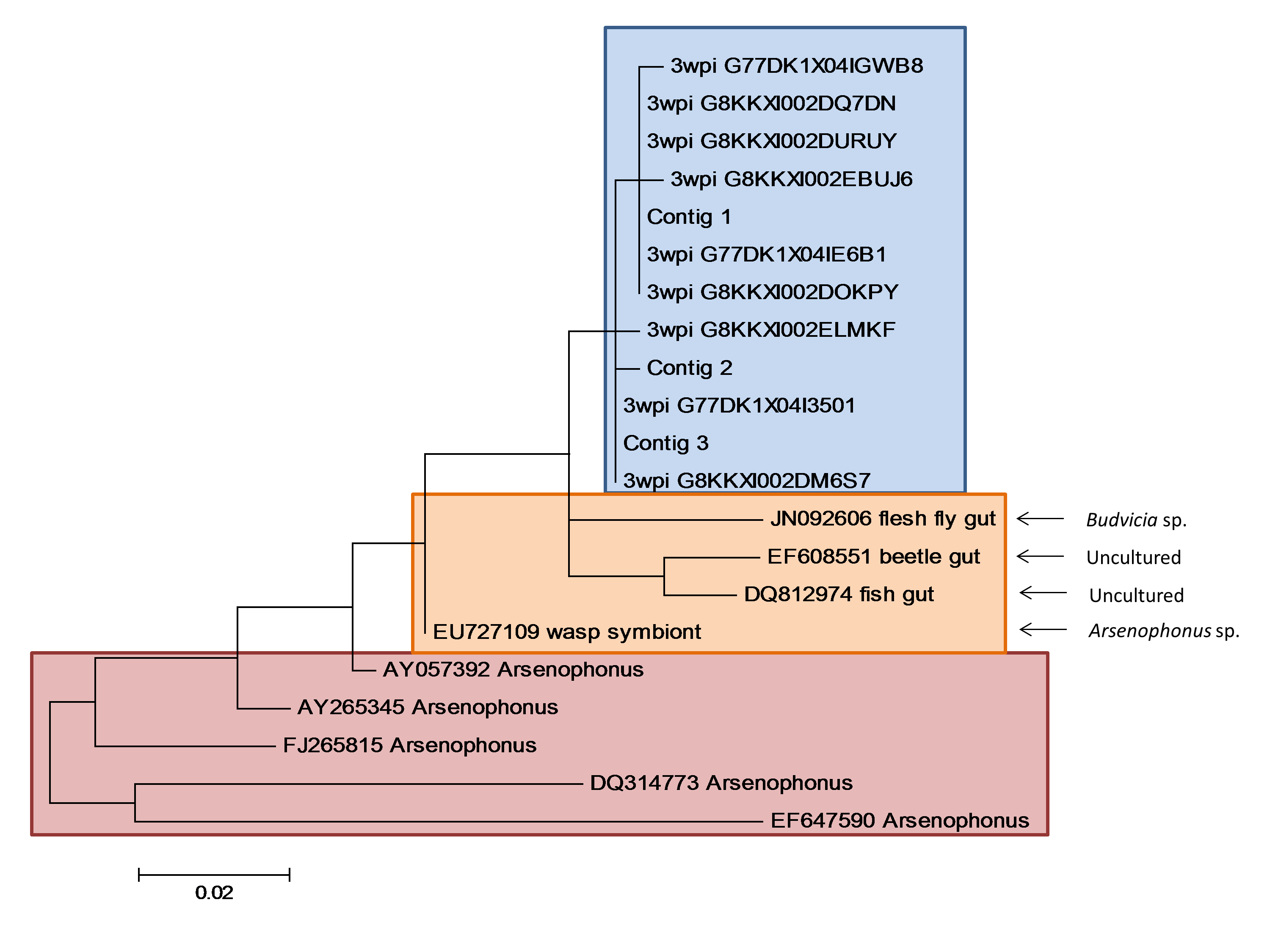

Supplement: Figure S1 — Molecular Phylogenetic analysis by Maximum Likelihood method of Rickettsia spp. The tree with the highest log likelihood (−1457.1543) is shown, and is drawn to scale, with branch lengths measured in the number of substitutions per site. The analysis involved 20 nucleotide sequences: reference Rickettsia samples from GenBank (red), sequences recovered from different life cycle stages of P. obamai n. sp. from this study (blue), and the 2 closest sequences recovered from GenBank BLAST hits (orange). All positions containing gaps and missing data were eliminated. There were a total of 305 positions in the final dataset. Clearly, the Rickettsia species known to induce parthenogenesis groups away from the sequences recovered as part of the current study and their closest BLAST match. Also note that these Rickettsia-like sequences were only recovered from two of the 5 life cycle stages tested. For additional information on sequences recovered by BLAST and contigs, see Table S1. (TIF) [file pone.0034472.s001.tif]

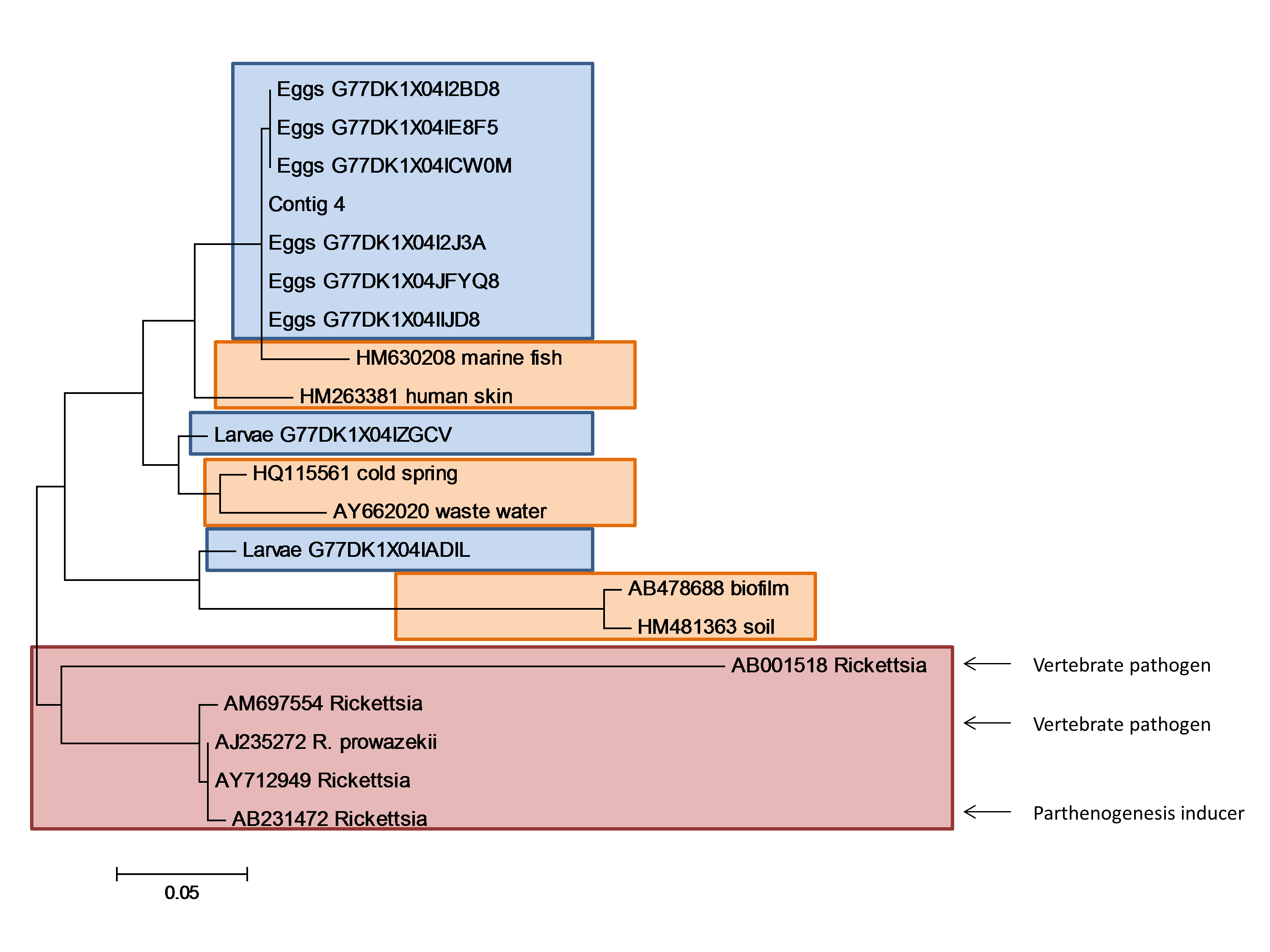

Supplement: Figure S2 — Molecular Phylogenetic analysis by Maximum Likelihood method of Arsenophonus spp. The tree with the highest log likelihood (−988.1006) is shown and is drawn to scale, with branch lengths measured in the number of substitutions per site. The analysis included 21 nucleotide sequences: reference Arsenophonus samples from GenBank (red), sequences recovered from 3 week old juvenile P. obamai n. sp. (blue), and the 2 closest sequences recovered from GenBank BLAST hits (orange) for each of the sequences recovered. All positions containing gaps and missing data were eliminated. There were a total of 321 positions in the final dataset. Note that the new sequences cluster with a sequence previously identified as Budvicia sp., as well as with symbionts known from wasps, not known to manipulate reproduction. Also note that these Arsenophonus-like sequences were only recovered from one of five life cycle stages tested. For additional information on sequences recovered by BLAST and contigs, see Table S1. (TIF) [file pone.0034472.s002.tif]

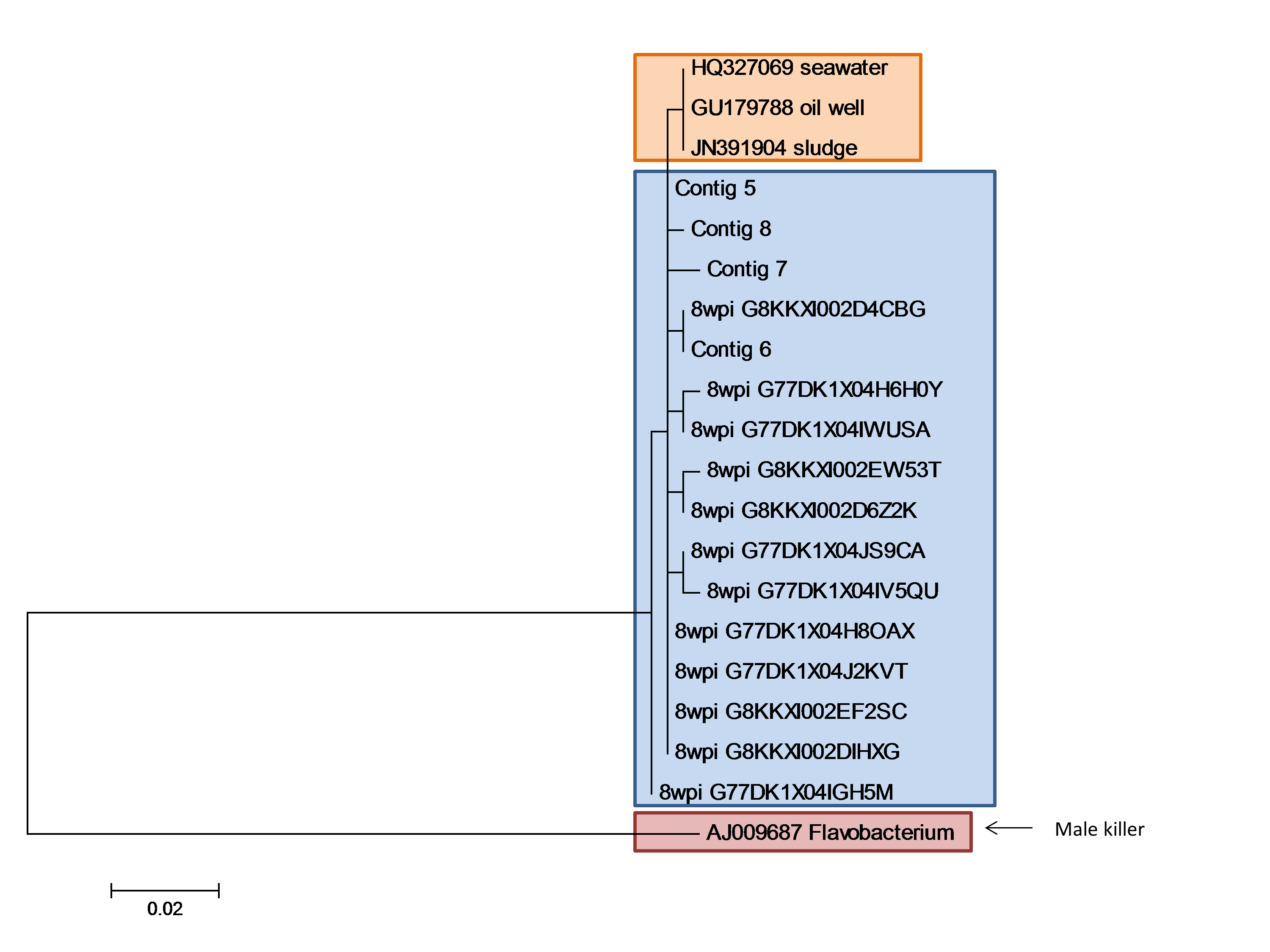

Supplement: Figure S3 — Molecular Phylogenetic analysis by Maximum Likelihood method of Flavobacteria. The tree with the highest log likelihood (−802.3099) is shown and is drawn to scale, with branch lengths measured in the number of substitutions per site. The analysis included 20 nucleotide sequences: reference Flavobacteria samples from GenBank (red), sequences recovered from 8 week old P. obamai n. sp. larvae (blue), and the 2 closest sequences recovered from GenBank BLAST hit (orange) for each sequence recovered. All positions containing gaps and missing data were eliminated. There were a total of 341 positions in the final dataset. Note that all new sequences and their closest BLAST matches fall into a separate group from the known Flavobacteria known to cause male killing. Also note that these Flavobacteria-like sequences were recovered from only one of five life cycle stages tested. For additional information on sequences recovered by BLAST and contigs, see Table S1. (TIF) [file pone.0034472.s003.tif]
