# Supplementary material for: Going Solo: Discovery of the First Parthenogenetic Gordiid (Nematomorpha: Gordiida)
Source: PLoS One. 2012 Apr 18;7(4):e34472. doi: 10.1371/journal.pone.0034472 (PMC3329510; doi:10.1371/journal.pone.0034472)
Supplement: Supplementary file 2 [file pone.0034472.s004.docx]

Table S1. Proportion of 16S bacterial rDNA sequences of different *Paragordius obamai* **n. sp.** life cycle stages falling into known categories of reproduction manipulation endosymbionts.

			P. obamai **n. sp.** life cycle stage				
**Endosymbiont group**	**Manipulation**	**Hosts**	**eggs**	**larvae**	**3wpi juv**	**8wpi juv**	**adult**
α-PROTEOBACTERIA							
Wolbachia pipientis	CI, F, PI, MK	Insecta, Nematoda	0	0	0	0	0
Rickettsia sp.	PI, MK	Insecta , Arachnida	0.10%	0.03%	0	0	0
Ɣ-PROTEOBACTERIA							
Arsenophonus sp.	MK	Insecta	0	0	1.04%	0	0
BACTEROIDETES							
Cardinium hertigii	CI, F, PI	Insecta, Arachnida	0	0	0	0	0
Flavobacterium sp.	MK	Insecta	0	0	0	0.93%	0
VERRUCOMICROBIA							
Xiphinematobacter	PI	Nematoda	0	0	0	0	0
TENERICUTES							
Spiroplasma sp.	MK	Insecta	0	0	0	0	0
		Total sequences analyzed	8291	7123	1915	2262	5201

3wpi juv: 3 week post infection juvenile worm, **8wpi juv**: 3 week post infection juvenile worm, **CI**: Cytoplasmic Incompatibility, **F**: Feminization, **PI**: Parthenogenesis induction, **MK**: Male killing
