# Supplementary material for: Going Solo: Discovery of the First Parthenogenetic Gordiid (Nematomorpha: Gordiida)
Source: PLoS One. 2012 Apr 18;7(4):e34472. doi: 10.1371/journal.pone.0034472 (PMC3329510; doi:10.1371/journal.pone.0034472)
Supplement: Supplementary file 3 [file pone.0034472.s005.docx]

Table S2. Sequences from 5 different *Paragordius obamai* **n. sp.** genomic DNA extractions with significant similarity to known genera of reproduction manipulating endosymbionts. Each DNA sequence was analyzed by BLAST, and the top 2 highest similarity hits (based pm e-values) were recorded.

TRL ID#	Sequence	ID	GB#	Source of Material	E-val
***Arsenophonus* sp.**					
>3wpi_Contig_1	TAATACGG/	Uncultured bacterium	EF608551	Gut of ground beetle	0
		Uncultured bacterium	DQ812974	Fish gut	0
>3wpi_Contig_2	TAATACGG/	Uncultured bacterium	EF608551	Gut of ground beetle	1.00E-116
		Arsenophonus symbiont	EU727109	Polistes nimpha (wasp)	7.00E-115
>3wpi_Contig_3	TAATACGG/	Uncultured bacterium	EF608551	Gut of ground beetle	5.00E-157
		Arsenophonus symbiont	EU727109	Polistes nimpha (wasp)	2.00E-155
>3wpi_G8KKXI002ELMKF	TAATACGG(	Arsenophonus symbiont	EU727109	Polistes nimpha (wasp)	0
		Uncultured bacterium	EF608551	Gut of ground beetle	0
>3wpi_G8KKXI002EBUJ6	TAATACGG/	Uncultured bacterium	EF608551	Gut of ground beetle	1.00E-158
		Arsenophonus symbiont	EU727109	Polistes nimpha (wasp)	5.00E-157
>3wpi_G8KKXI002DURUY	TAATACGG/	Arsenophonus symbiont	EU727109	Polistes nimpha (wasp)	4.00E-178
		Uncultured bacterium	EF608551	Gut of ground beetle	1.00E-174
>3wpi_G8KKXI002DQ7DN	TAATACGG/	Uncultured bacterium	EF608551	Gut of ground beetle	2.00E-115
		Arsenophonus symbiont	EU727109	Polistes nimpha (wasp)	8.00E-114
>3wpi_G8KKXI002DOKPY	TAATACGG/	Arsenophonus symbiont	EU727109	Polistes nimpha (wasp)	2.00E-177
		Uncultured bacterium	EF608551	Gut of ground beetle	3.00E-174
>3wpi_G8KKXI002DM6S7	TAATACGG/	Arsenophonus symbiont	EU727109	Polistes nimpha (wasp)	2.00E-62
		Budvicia sp.	JN092606	Gut of flesh fly	1.00E-60
>3wpi_G77DK1X04IGWB8	TAATACGG/	Uncultured bacterium	EF608551	Gut of ground beetle	4.00E-132
		Arsenophonus symbiont	EU727109	Polistes nimpha (wasp)	2.00E-130
>3wpi_G77DK1X04IE6B1	CGGTAATA(	Arsenophonus symbiont	EU727109	Polistes nimpha (wasp)	1.00E-152
		Uncultured bacterium	EF608551	Gut of ground beetle	2.00E-151
>3wpi_G77DK1X04I3501	CGGTAATA(	Arsenophonus symbiont	EU727109	Polistes nimpha (wasp)	1.00E-173
		Uncultured bacterium	EF608551	Gut of ground beetle	3.00E-170
Rickettsia sp.					
>Eggs_G77DK1X04ICW0M	TAATACGG/	Uncultured Bacterium	HM630208	Gut of marine fish	0
		Environmental Sample	HM263381	Human skin	5.00E-163
>Eggs_G77DK1X04IE8F5	TAATACGG/	Uncultured Bacterium	HM630208	Gut of fish	3.00E-175
		Environmental Sample	HM263381	Human skin	1.00E-154
>Eggs_G77DK1X04IIJD8	TAATACGG/	Uncultured Bacterium	HM630208	Gut of fish	5.00E-162
		Environmental Sample	HM263381	Human skin	4.00E-143
>Eggs_G77DK1X04JFYQ8	TAATACGG/	Uncultured Bacterium	HM630208	Gut of fish	2.00E-136
		Environmental Sample	HM263381	Human skin	4.00E-123
>Eggs_G77DK1X04I2J3A	TAATACGG/	Uncultured Bacterium	HM630208	Gut of fish	3.00E-139
		Environmental Sample	HM263381	Human skin	6.00E-126
>Eggs_G77DK1X04I2BD8	TAATACGG/	Uncultured Bacterium	HM630208	Gut of fish	7.00E-105
		Environmental Sample	HM263381	Human skin	2.00E-91
>Eggs_Contig_4	TAATACGG/	Uncultured Bacterium	HM630208	Gut of fish	8.00E-145
		Environmental Sample	HM263381	Human skin	8.00E-130
>Larvae_G77DK1X04IADIL	TAATACGG/	Environmental Sample	HM481363	TCE contaminated field site	2.00E-111
		Environmental Sample	AB478688	biofilm mats of a montane wetland in the Alps	1.00E-109
>Larvae_G77DK1X04IZGCV	TAATACGG/	Environmental Sample	HQ115561	Cold spring	8.00E-140
		Environmental Sample	AY662020	Uranium waste water	3.00E-124

***Flavobacterium* sp.**					
>8wpi_Contig_5	TAATACGG/	Environmental sample	GU179788	Alaska, North Slope	7.00E-109
		Environmental sample	HQ327069	Seawater	7.00E-109
>8wpi_Contig_6	TAATACGG/	Environmental sample	GU179788	Alaska, North Slope	2.00E-119
		Environmental sample	HQ327069	Seawater	2.00E-119
>8wpi_Contig_7	TAATACGG/	Environmental sample	GU179788	Alaska, North Slope	2.00E-104
		Environmental sample	HQ327069	Seawater	2.00E-104
>8wpi_Contig_8	TAATACGG/	Environmental sample	GU179788	Alaska, North Slope	2.00E-104
		Environmental sample	HQ327069	Seawater	2.00E-104
>8wpi_G8KKXI002EF2SC	TAATACGG/	Environmental Sample	HQ327069	seawater	9.00E-98
		Environmental Sample	GU179788	oil well, AK	9.00E-98
>8wpi_G8KKXI002DIHXG	TAATACGG/	Environmental Sample	GU179788	oil well, AK	0
		Environmental Sample	JN391904	sludge in anoxic tank	0
>8wpi_G8KKXI002EW53T	TAATACGG/	Environmental Sample	GU179788	oil well, AK	0
		Environmental Sample	JN391904	sludge in anoxic tank	0
>8wpi_G8KKXI002D6Z2K	TAATACGG/	Environmental Sample	GU179788	oil well, AK	3.00E-174
		Environmental Sample	JN391904	sludge in anoxic tank	3.00E-174
>8wpi_G8KKXI002D4CBG	TAATACGG/	Environmental Sample	GU179788	oil well, AK	3.00E-159
		Environmental Sample	JN391904	sludge in anoxic tank	3.00E-159
>8wpi_G77DK1X04IGH5M	TAATACGG/	Environmental Sample	GU179788	oil well, AK	4.00E-178
		Environmental Sample	JN391904	sludge in anoxic tank	4.00E-178
>8wpi_G77DK1X04IWUSA	TAATACGG/	Environmental Sample	GU179788	oil well, AK	4.00E-178
		Environmental Sample	JN391904	sludge in anoxic tank	4.00E-178
>8wpi_G77DK1X04IV5QU	CAATACGG.	Environmental Sample	GU179788	oil well, AK	1.00E-173
		Environmental Sample	JN391904	sludge in anoxic tank	1.00E-173
>8wpi_G77DK1X04H8OAX	TAATACGG/	Environmental sample	GU179788	Alaska, North Slope	2.00E-166
		Environmental sample	HQ327069	Seawater	2.00E-166
>8wpi_G77DK1X04JS9CA	TAATACGG/	Environmental Sample	GU179788	oil well, AK	1.00E-162
		Environmental Sample	JN391904	sludge in anoxic tank	1.00E-162
>8wpi_G77DK1X04H6H0Y	TAATACGG/	Environmental sample	GU179788	Alaska, North Slope	9.00E-98
		Environmental sample	HQ327069	Seawater	9.00E-98
>8wpi_G77DK1X04J2KVT	TAATACGG/	Environmental Sample	GU179788	oil well, AK	3.00E-164
		Environmental Sample	JN391904	sludge in anoxic tank	3.00E-164
**Contig members**					
>3wpi_Contig_1:	>3wpi_G77DK1X04JC3MY				
	>3wpi_G8KKXI002D5RCM				
	>3wpi_G8KKXI002ET1OX				
	>3wpi_G8KKXI002DFLA8				
	>3wpi_G8KKXI002DA7SL				
	>3wpi_G8KKXI002ENYVV				
	>3wpi_G8KKXI002D50TQ				
>3wpi_Contig_2	>3wpi_G8KKXI002C9MDM				
	>3wpi_G8KKXI002DR3M2				
>3wpi_Contig_3	>3wpi_G8KKXI002DOJLZ				
	>3wpi_G8KKXI002C35F1				
>3wpi_Contig_4	>Eggs_G77DK1X04H5KJS				
	>Eggs_G77DK1X04IN24W				

>8wpi_Contig_5	>8wpi_G77DK1X04JZ8YC
	>8wpi_G8KKXI002C1YP8
	>8wpi_G8KKXI002D1TGT
	>8wpi_G77DK1X04I08K1
>8wpi_Contig_6	>8wpi_G77DK1X04H2AMT
	>8wpi_G77DK1X04JMUJJ
>8wpi_Contig_7	>8wpi_G8KKXI002DTOMC
	>8wpi_G77DK1X04H49O5
>8wpi_Contig_8	>8wpi_G8KKXI002CZXP1
	>8wpi_G77DK1X04H9W6R
